# Importance of early treatment decisions on future income of multiple sclerosis patients

**DOI:** 10.1177/2055217320959116

**Published:** 2020-10-07

**Authors:** Andrius Kavaliunas, Ali Manouchehrinia, Hanna Gyllensten, Kristina Alexanderson, Jan Hillert

**Affiliations:** Department of Clinical Neuroscience, Karolinska Institutet, Stockholm, Sweden; Department of Clinical Neuroscience, Karolinska Institutet, Stockholm, Sweden; Centre for Person-Centred Care (GPCC), and Institute of Health and Care Sciences, Sahlgrenska Academy, University of Gothenburg, Gothenburg, Sweden; Department of Clinical Neuroscience, Karolinska Institutet, Stockholm, Sweden; Department of Clinical Neuroscience, Karolinska Institutet, Stockholm, Sweden; Karolinska University Hospital, Stockholm, Sweden

**Keywords:** Multiple sclerosis, drug therapy, time-to-treatment, income, socioeconomic factors, sick leave, cohort studies

## Abstract

**Background:**

Early initiation of disease-modifying treatment (DMT) is associated with better disability outcomes in multiple sclerosis (MS). However, little is known of how treatment decisions affect socio-economic outcomes.

**Objective:**

To estimate the long-term impact of early initiation of DMT on the income of MS patients.

**Methods:**

In total, 3610 MS patients were included in this register-based cohort study. We measured the association between the time to treatment and the outcome, defined as time from treatment initiation to a 95% decrease in annual earnings compared to each patient´s baseline level. Additionally, the association between time to treatment and increase of social benefits (sickness absence, disability pension) was investigated. A Cox model was adjusted for sex, onset age, education, family situation, country of birth, living area, and disability.

**Results:**

MS patients initiating treatment later had a higher risk of reaching the outcome- those who started treatment after 2 years from MS onset lost 95% of their earnings sooner (HR, 1.19; 95% CI, 1.04–1.37). Furthermore, risk to receive an annual compensation of SEK 100,000 (≈EUR 10,500) was higher for the delayed treatment group.

**Conclusion:**

Early treatment initiation in MS is associated with better socioeconomic outcome, adding to previous studies showing benefits regarding disability.

## Introduction

Multiple sclerosis (MS) continues to be a challenging and disabling condition, predominantly affecting individuals in their early life, and has an impact functionally, financially, and on quality of life.^1^ Recent years have seen a large expansion in the therapeutic options for MS.^2,3^ The emergence of effective disease-modifying treatments (DMT) has created an impetus to diagnose as early as possible and the plethora of new agents poses challenges in selecting the right drug for the right person at the right time.^1,4^

According to the current guidelines, DMT should be available to all people with relapsing forms of MS,^5^ and should be offered as early as possible^2^ as early treatment initiation is associated with better physical outcomes, both in the short- and long-term.^3,6–8^ Although there is a clear association between health and income,^9^ and MS is associated with lower productivity at work, and higher levels of sickness absence and disability pension,^10–12^ little is known of how treatment decisions affect socio-economic outcomes in MS patients.

The aim of this study was to estimate the long-term impact of early treatment initiation on the income of MS patients.

## Materials and methods

### Study design

We conducted an observational cohort study with retrospective analysis on prospectively collected data to assess the impact of early treatment initiation on income of MS patients. The patients who started DMT during 2001 – 2012 were included in the study and followed-up through 2013. The following inclusion criteria were also used: 1) patient´s age: 18-64 years old at inclusion and during the follow-up period (to be at risk for the study outcomes; due to the retirement age); 2) no missing values (7 patients were not included due to missing age at onset, 6 – due to missing education information, and 9 – due to missing family situation) in the variables used for the analyses, as this is a prerequisite for multivariate regression (described below).

Microdata from two Swedish nationwide registers were linked at individual level using the unique personal identification number assigned to all residents in Sweden. The clinically generated Swedish Multiple Sclerosis Register^13,14^ – which is used in all neurology departments in the country and currently includes data on 19,620 patients (∼80% of Sweden´s estimated prevalent MS patients) – was utilized to obtain information about individuals diagnosed with MS, including their age at clinical MS onset (the first reported clinical symptoms), the baseline scores of the Expanded Disability Status Scale (EDSS), and DMT initiation. The following DMTs were included: interferon beta, glatiramer acetate, natalizumab, fingolimod, rituximab, teriflunomide, alemtuzumab, mitoxantrone, and dimethyl fumarate.

The Longitudinal Integration Database for Health Insurance and Labor Market Studies (LISA), held by Statistics Sweden, was used for patient-level information on their annual income and socio-demographic variables (sex, age, family situation, type of living area, educational level, and country of birth).^15^

Lastly, for the purpose of comparison and interpretation of the results, patients were stratified into two treatment groups by the time to treatment initiation, set as initiation of the first DMT within two years (≤24 months) and after two years (>24 months) from MS onset.

## Outcomes

The study outcome was defined as time from treatment initiation to a 95% decrease in annual earnings in Swedish Crowns (SEK) compared to each patients’ baseline level, i.e. at treatment initiation. The choice of an analysis for such a significant decrease was guided by the fluctuating nature of the income (i.e., low levels of decrease are expected). Additionally, the association between time to treatment and increase of financial compensations from social security systems, i.e., social benefits, by SEK 100,000 was examined. These five sources of incomes were combined and analyzed as ´benefits´: disability pension, sickness absence, disability allowance, unemployment compensation, and social assistance.^15^

### Statistical analyses

Descriptive statistics with means, medians, and proportions were used to describe the study population at baseline. One-way analysis of variance (ANOVA) was used to compare means of continuous variables between the two treatment groups; to compare medians of ordinal variables, a non-parametric Kruskal-Wallis test was used. For the categorical variables, a Chi-square test was used. Differences were defined as statistically significant for p values lower than 0.05.

A survival analysis was used to measure the association between the time from MS onset to treatment initiation and the study outcomes. The crude and adjusted hazard ratios (HR) and their 95% confidence intervals (CI) were estimated using Cox regression. The models were adjusted for sex, age at MS onset, educational level, family situation (dichotomized into being married, living with a partner (cohabitant) vs. living single), country of birth, type of living area, and baseline EDSS. The proportional hazard assumption underlying the analysis was analyzed graphically by creating log–log plots and on the basis of Schoenfeld residuals. Product terms between variables were checked for interaction using likelihood ratio test and comparing the models. Time to treatment initiation was also studied using Cox regression model adjusted for propensity score. Propensity score was calculated based on sex, age at MS onset, education, country of birth, type of living area, and baseline EDSS.

## Ethics

The project was approved by the Regional Ethical Review Board of Stockholm. All patients gave written informed consent for their data to be included in the Swedish MS Register.

## Results

### Analysis of earnings

The total number of patients included in the analysis was 3610. The median follow-up time was four years (the interquartile range: 2 to 7; the maximum possible value 12); total analysis time at risk was 16,888 years. More than two-thirds of the patients were females; the mean age at MS clinical onset was 32.6 years, and the median baseline EDSS score was 1.5 (Table 1). Also, a majority of the patients were born in the European Union countries (or Norway) and had at least secondary education, about half were married or cohabitant.

**Table 1.** Clinical and demographic characteristics of the study population in the analysis of earnings.

Patients’ characteristics	All patients	Time to treatment		p-value
		≤2 years	>2 years	
Number of patients	3610 (100%)	2133 (59%)	1477 (41%)	–
Sex:				0.7^a^
Males	1073 (30%)	628 (29%)	445 (30%)	
Females	2537 (70%)	1505 (71%)	1032 (70%)	
Age at MS onset (mean (SD))	32.6 (9.7)	34.1 (9.8)	30.4 (9.2)	<0.001^b^
Age at treatment initiation (mean (SD))	37.5 (10.2)	35.1 (9.8)	41.0 (9.9)	<0.001^b^
Baseline EDSS (median (IQR))	1.5 (1.5)	1.5 (1.5)	2 (2)	<0.001^c^
Education:				0.2^a^
Higher	1585 (44%)	915 (43%)	670 (45%)	
Secondary	1730 (48%)	1049 (49%)	681 (46%)	
Lower	295 (8%)	196 (8%)	126 (9%)	
Family situation:				<0.001^a^
Married/cohabitant	1914 (53%)	999 (47%)	915 (62%)	
Single	1696 (47%)	1134 (53%)	562 (38%)	
Country of birth:				0.7^a^
EU and Norway	3423 (95%)	2020 (95%)	1403 (95%)	
Other	187 (5%)	113 (5%)	74 (5%)	
Type of living area:				0.5^a^
Larger cities	1,643 (46%)	966 (45%)	677 (46%)	
Medium-sized municipalities	1127 (31%)	683 (32%)	444 (30%)	
Smaller municipalities	840 (23%)	484 (23%)	356 (24%)	
Number of patients who reached the outcome	849 (24%)	435 (20%)	414 (28%)	<0.001^a^

p-value: for comparisons between two time to treatment groups (≤2 years vs. >2 years); SD: standard deviation; IQR: interquartile range; EDSS: Expanded Disability Status Scale; EU: the European Union.

^a^Chi-square test.

^b^One-way ANOVA.

^c^Kruskal-Wallis test.

We can notice several differences when comparing the two treatment groups. First of all, MS patients in the delayed treatment group (time to treatment >2 years) had on average earlier MS onset (30.4 years vs. 34.1 years; p < 0.001), they were slightly more disabled (the median EDSS score of 2 vs. 1.5; p < 0.001) and displayed a higher proportion of married or cohabitant (62% vs. 47%; p < 0.001). On the other hand, proportions regarding sex, educational level, country of birth, and type of living area were similar in the two groups.

The initial univariate Cox regression analysis showed a statistically significant HR of 1.27 (95% CI, 1.11–1.45) (Supplementary Table 1, Supplementary Figure 1). It also showed that late MS onset, lower educational level, being single, being born outside the EU countries (or Norway), and higher baseline disability, but not sex or type of living area, were associated with the outcome.

**Figure 1.**
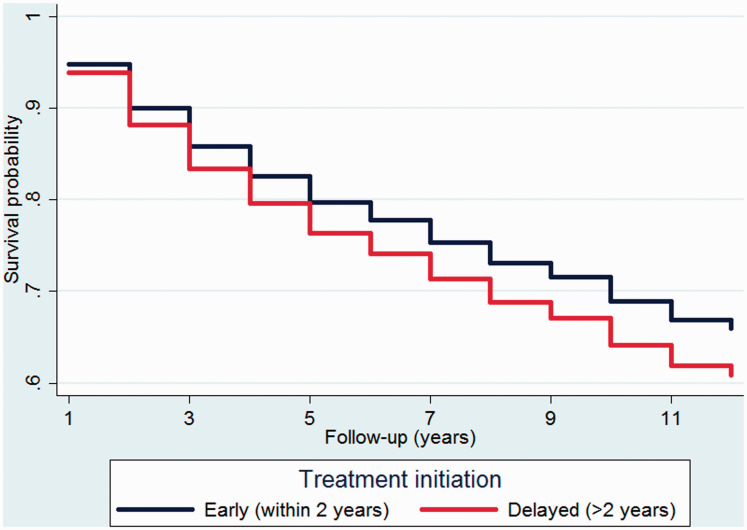
A survivor function plotted for the two treatment groups after fitting a Cox model and adjusting for the covariates.

The higher risk to lose earnings within the delayed treatment group remained statistically significant and was associated with a worse outcome after adjusting for the covariates (Table 2 and Figure 1). Thus, MS patients who started treatment after two years from MS onset lost 95% of the earnings sooner with the adjusted HR of 1.19 (95% CI, 1.04–1.37). Additionally, HR for females increased from 1.03 (95% CI, 0.89–1.20) in crude analysis to 1.22 (95% CI, 1.05–1.42) in the adjusted analysis and now turned to be significantly associated with the outcome. All other covariates retained the significance level and directionality (type of living area remained insignificant).

**Table 2.** Adjusted hazard ratios to lose earnings.

Covariate	HR	SE	p	95% CI	
Time to treatment:					
≤2 years	Ref.				
>2 years	1.19	0.09	0.015	1.04	1.37
Sex:					
Males	Ref.				
Females	1.22	0.09	0.008	1.05	1.42
Age at onset:					
<50 years	Ref.				
≥50 years	1.74	0.22	<0.001	1.36	2.23
Education:					
Higher	Ref.				
Secondary	1.95	0.16	<0.001	1.67	2.28
Lower	2.79	0.31	<0.001	2.24	3.48
Family situation:					
Married/cohabitant	Ref.				
Single	1.36	0.09	<0.001	1.18	1.56
Country of birth:					
EU and Norway	Ref.				
Other	1.88	0.25	<0.001	1.44	2.45
Type of living area:					
Larger cities	Ref.				
Medium-sized municipalities	0.97	0.08	0.73	0.83	1.14
Smaller municipalities	1.08	0.09	0.38	0.91	1.28
Baseline EDSS:					
0–1.5	Ref.				
2–4.5	1.68	0.13	<0.001	1.44	1.95
≥5	4.33	0.50	<0.001	3.45	5.42

HR: hazard ratio; SE: standard error; CI: confidence intervals; Ref.: reference; EU: the European Union; EDSS: Expanded Disability Status Scale.

### Analysis of benefits

For this analysis we used another outcome – sum of the five available social benefits. The number of patients included in this analysis was 2975. The median follow-up time was four years (the interquartile range: 2 to 6). The baseline demographic and clinical characteristics of the patients were similar to those already reported for the above analysis (Supplementary Table 2), and the structure of the constituting benefits sources was similar to those previously reported,^16,17^ with a greater part (82%) of the total annual benefits comprised from disability pension and sickness absence.

The univariate Cox regression analysis showed that delay of treatment initiation increased the risk to receive benefits by 42% (HR, 1.42; 95% CI, 1.23–1.65). It also showed that lower educational level, living in a smaller municipality, being born outside the EU countries, and higher baseline disability, but not sex, family situation or MS onset age were associated with the outcome (Supplementary Table 3).

The higher risk to receive benefits within the delayed treatment group remained statistically significant after adjusting for the covariates (Table 3). Thus, MS patients who started treatment after two years from MS onset received the amount of SEK 100,000 benefits sooner (HR, 1.23; 95% CI, 1.05–1.43). Additionally, HR for females increased from 1.16 (95% CI, 0.98–1.37) in crude analysis to 1.24 (95% CI, 1.05–1.47) in the adjusted analysis and now turned to be significantly associated with the outcome. All other covariates retained at similar significance level and directionality (MS onset age and family situation remained insignificant).

**Table 3.** Adjusted hazard ratios to receive benefits.

Covariate	HR	SE	p	95% CI	
Time to treatment:					
≤2 years	Ref.				
>2 years	1.23	0.10	0.01	1.05	1.43
Sex:					
Males	Ref.				
Females	1.24	0.11	0.01	1.05	1.47
Age at onset:					
<50 years	Ref.				
≥50 years	1.12	0.19	0.5	0.80	1.57
Education:					
Higher	Ref.				
Secondary	1.28	0.11	0.003	1.09	1.51
Lower	1.63	0.20	<0.001	1.29	2.07
Family situation:					
Married/cohabitant	Ref.				
Single	1.01	0.19	0.85	0.87	1.18
Country of birth:					
EU and Norway	Ref.				
Other	1.50	0.21	0.003	1.14	1.98
Type of living area:					
Larger cities	Ref.				
Medium-sized municipalities	1.32	0.11	0.001	1.11	1.56
Smaller municipalities	1.31	0.13	0.007	1.07	1.59
Baseline EDSS:					
0–1.5	Ref.				
2–4.5	1.98	0.16	<0.001	1.69	2.32
≥5	4.64	0.63	<0.001	3.56	6.04

HR: hazard ratio; SE: standard error; CI: confidence interval; Ref.: reference; EU: the European Union; EDSS: Expanded Disability Status Scale.

### Subgroup and sensitivity analyses

As our treatment groups were categorized by time to treatment arbitrarily, we additionally investigated alternative categorizations of this time period. E.g., time to treatment within one year, one to three years, and more than three years (i.e., three categories, also used by us previously^6^) yielded a similar HR of 1.22 (95% CI, 1.05–1.42) to lose earnings and HR of 1.26 (95% CI, 1.07–1.48) to receive benefits within the delayed treatment group (>3 years) when compared to the early treatment group (within one year); whereas the middle group (one to three years) did not differ (in both earnings and benefits analyses), in fact justifying our main analysis approach to collapse this group. Further categorization into four groups (0–6, 7–12, 13–36, and 36+ months) also showed that the most delayed treatment group (36+ months) had a significantly higher risk to reach the outcomes – HR was 1.23 (95% CI, 1.02–1.48) to lose earnings and HR of 1.30 (95% CI, 1.08–1.58) to receive benefits. Furthermore, a model with uncategorized time to treatment as continuous variable (in years) showed the HRs of 1.02 (95% CI, 1.01–1.03, p < 0.001) in both earnings and benefits analyses, meaning that each year of treatment delay increased the risk of the outcomes by 2%.

Similarly, we conducted additional analyses examining alternative levels of earnings decrease, e.g., 70%, 80%, 90%, and 100% that yielded similar HRs of 1.13 (95% CI, 1.00 – 1.28), 1.17 (1.03–1.33), 1.20 (1.05–1.37), 1.29 (1.11–1.50), respectively. One can notice an increasing tendency of the hazard together with the increasing level of lost earnings, thus the lower levels (e.g., 10%, 20%, 30%) were not significantly associated with the outcome – which is not surprising, given the fluctuating nature of the income. Analyses with the alternative levels of the benefits received also confirmed the main results, e.g., HRs were 1.22 (95% CI, 1.04–1.42) and 1.22 (95% CI, 1.05–1.41) to receive annual benefits of SEK 10,000 and SEK 50,000, respectively.

To support our findings, we additionally applied propensity score analysis. The adjusted for propensity score HRs within the delayed treatment group were 1.17 (95% CI, 1.02–1.34) and 1.23 (95% CI, 1.06–1.43) for loosing earnings and receiving benefits respectively – in line with our main results.

To appreciate the complexity of the clinical course of MS and our chosen outcomes, which both require relatively long time horizon for the analysis, as well as to give enough time to ascertain the correct categorization of the treatment groups (i.e. avoid the untreated patients to become delayed treatment group at some time point), we additionally analyzed the subgroup of the patients with the longest follow-up (at least 6 years), which resulted in significantly higher risk to lose earnings within the delayed treatment group (HR, 1.67; 95% CI, 1.17–2.45), but not the risk to receive the benefits (HR, 1.34; 95% CI, 0.86–2 .07). Interestingly, in these analyses sex was not significantly associated with the outcomes (HRs 1.03 and 1.14; p > 0.5; for earnings and benefits, respectively).

## Discussion

In this register-based cohort study we investigated how early or delayed treatment initiation was associated with the income of MS patients. We found that patients initiating treatment later had a higher risk of reaching the unfavorable outcome, e.g., those who started treatment after 2 years from MS onset lost 95% of their earnings sooner with the adjusted HR of 1.19 (95% CI, 1.04–1.37). Furthermore, risk to receive a certain amount of income from the social benefits (e.g., sickness absence, disability pension) was higher for the delayed treatment group (e.g., the adjusted HR to reach an annual compensation of SEK 100,000 (≈EUR 10,500) for those who started treatment later was 1.23 (95% CI, 1.05–1.43)).

To our knowledge, this is the first study to investigate the initiation of treatment in the context of income of MS patients. Our results are in line with a study confirming the benefits of early treatment with regards to the risk of disability pension – MS patients initiating treatment early had a 36% lower risk of full-time disability pension.^18^ It is also in line with our recent study, highlighting a sharp increase of net days of sickness absence and disability pension over time in the period around diagnosis.^12^ Some other studies^19,20^ also indicated a much lower income among MS patients when compared to the general population; and a study in Denmark showed that the probability of remaining without early retirement at 5 years decreased by 30% in MS patients.^21^

Besides investigating the benefits of early treatment initiation, we could also illustrate the impact of other factors, particularly sex. The role of sex in the epidemiology of MS is an obvious topic given the higher risk of MS among females,^22^ and studies often find males to be associated with a less favorable outcome in terms of progression to disability landmarks.^23^ In contrast, we show that female sex was associated with less favorable outcome (i.e., 22% higher risk to lose earnings), however, this is not surprising given the socioeconomic nature of our outcomes, as in general female sex is associated with lower salaries. Interestingly, sex was not a significant factor in both crude analyses of earnings and benefits but turned to be significant after adjusting for other covariates. Also, a similar phenomenon was noticed in a subgroup analysis of the patients with the longest follow-up time (sex was not significantly associated with the outcomes). Previously, we also saw such a varying significance in the context of physical disability outcomes – males had a higher risk for progression, but only for the long-term disability milestones, such as EDSS 6 (and not, e.g., EDSS 4).^6^ Clearly, these aspects could be well investigated further for a more definitive answer.

Such factors as female sex, lower educational level, being born outside Sweden, or living in smaller municipalities were also shown to be associated with a higher risk for disability pension^24,25^ and lower risk to receive earnings, as well as lower levels of earnings.^16^ Notably, these factors also bore higher risk estimates than our main exposure variable, however, when it comes to a risk modification and disease management in clinical practice, treatment is usually among the most important interventions.

One could also hypothesize that the impact of various factors on the risk to lose earnings and receive benefits is similar, or at least works in the same direction. In our study this was true for time to treatment, sex, education, country of birth, and baseline disability. However, age at onset and family situation, though being in the same directionality, were not significant for the benefits, while, interestingly, the type of living area was. This could be explained by the fact that generally individuals’ earnings depend on a variety of different factors, like age, education, labor market experience, etc., but also such seemingly irrelevant personal characteristics, like beauty, height, obesity.^26^ Apparently, the social security system is more fair, as such factors like onset age or family situation did not play a significant role for the risk to receive benefits.

The strengths of our study include a large sample and the population-based register approach, linking microdata from two databases, enabling use of sociodemographic and clinical data of high quality.^27^ Undoubtedly, an important advantage of this study is the possibility to adjust the estimates of MS patients´ income for a number of sociodemographic variables, like age, sex, educational level, family situation, type of living area, country of birth, as well as for important clinical data, like EDSS and MS onset age. A given limitation of this study is a lack of information about other factors, both environmental and genetic, possibly associated with the clinical course and outcomes of the disease (e.g., smoking, pregnancy), which we could not address in this study. Also, information about the clinical onset of MS is collected retrospectively, and thus might be subjected to recall bias. Finally, the observational nature of our data and study design also limits the implications, as it is not possible to infer the causality for the identified associations. However, besides exploring the novel outcomes to study disease progression, our study also includes several additional analyses to ascertain our findings, including different categorization of the outcomes and the main exposure (i.e., time to treatment), and a subgroup analysis of the longest surviving patients – that are all in line with the main results. As the patients in our treatment groups have several differences in the baseline characteristics, we also applied a propensity score analysis, which is suggested as a proper tool to mitigate selection bias – one of the main limitation in observational studies – of course, to a limit of the measured confounders.^28^ The latter model also supported our findings. However, confirmation in future studies is important, including study designs allowing stratification between first- and second-line treatments.

An underlying purpose of the study was also to illustrate how income, as an outcome, can be used to study clinical progression of MS. In a number of studies we have already shown how income highly correlates with physical disability and reflects the clinical course, e.g., increasing disability was associated with higher chance to receive social benefits and with lower chance to have earnings;^16^ primary and secondary progressive MS patients were similar from the perspective of patients´ income and sickness absence/disability pension, while relapsing remitting MS patients proved to have much higher earnings, less benefits, and lower levels of sickness absence and disability pension than the two other groups.^29,30^ Moreover, lower cognitive function affects the financial situation of MS patients negatively and independently of physical disability.^31^ In contrast to clinical scores such as EDSS, which are collected irregularly in the real world setting, socioeconomic data, when available as in Sweden, offer measures with no data loss, i.e., for all periods for all patients. Besides overcoming the ever-present challenge with missing data in observational studies, income data can encompass other aspects of the disease, such as fatigue and cognition, not captured by physical disability. In conclusion, we confirm the benefits of early treatment initiation in the socioeconomic context using the novel and unbiased outcome, also support the idea that it can serve as a precise outcome measure and can be used as a proxy parameter of disability.

## Supplemental Material

sj-pdf-1-mso-10.1177_2055217320959116 - Supplemental material for Importance of early treatment decisions on future income of multiple sclerosis patients Click here for additional data file. Supplemental material, sj-pdf-1-mso-10.1177_2055217320959116 for Importance of early treatment decisions on future income of multiple sclerosis patients by Andrius Kavaliunas, Ali Manouchehrinia Hanna Gyllensten Kristina Alexanderson Jan Hillert in Multiple Sclerosis Journal—Experimental, Translational and Clinical

sj-pdf-2-mso-10.1177_2055217320959116 - Supplemental material for Importance of early treatment decisions on future income of multiple sclerosis patients Click here for additional data file. Supplemental material, sj-pdf-1-mso-10.1177_2055217320959116 for Importance of early treatment decisions on future income of multiple sclerosis patients by Andrius Kavaliunas, Ali Manouchehrinia Hanna Gyllensten Kristina Alexanderson Jan Hillert in Multiple Sclerosis Journal—Experimental, Translational and Clinical

## References

[1] Thompson AJ Baranzini SE Geurts J, et al Multiple sclerosis. Lancet 2018; 391: 1622–1636. 29576504 10.1016/S0140-6736(18)30481-1

[2] Montalban X Gold R Thompson AJ, et al ECTRIMS/EAN guideline on the pharmacological treatment of people with multiple sclerosis. Mult Scler 2018; 24: 96–120. 29353550 10.1177/1352458517751049

[3] Cerqueira JJ Compston DAS Geraldes R, et al Time matters in multiple sclerosis: can early treatment and long-term follow-up ensure everyone benefits from the latest advances in multiple sclerosis? J Neurol Neurosurg Psychiatry 2018; 89: 844–850. 29618493 10.1136/jnnp-2017-317509 PMC6204938

[4] Gafson A Craner MJ Matthews PM. Personalised medicine for multiple sclerosis care. Mult Scler 2017; 23: 362–369. 27672137 10.1177/1352458516672017

[5] Rae-Grant A Day GS Marrie RA, et al Practice guideline recommendations summary: disease-modifying therapies for adults with multiple sclerosis: report of the guideline development, dissemination, and implementation subcommittee of the American academy of neurology. Neurology 2018; 90: 777–788. 29686116 10.1212/WNL.0000000000005347

[6] Kavaliunas A Manouchehrinia A Stawiarz L, et al Importance of early treatment initiation in the clinical course of multiple sclerosis. Mult Scler 2017; 23: 1233–1240. 27754943 10.1177/1352458516675039

[7] Chalmer TA Baggesen LM Nørgaard M, et al; the Danish Multiple Sclerosis Group. Early versus later treatment start in multiple sclerosis: a register-based cohort study. Eur J Neurol 2018; 25: 1262–e1110. 10.1111/ene.13692 29847005

[8] Sorensen PS. New management algorithms in multiple sclerosis. Curr Opin Neurol 2014; 27: 246–259. 2014/04/25. 24759080 10.1097/WCO.0000000000000096

[9] Fritzell J Nermo M Lundberg O. The impact of income: assessing the relationship between income and health in Sweden. Scand J Public Health 2004; 32: 6–16. 14757543 10.1080/14034950310003971

[10] Landfeldt E Castelo-Branco A Svedbom A, et al Sick leave and disability pension before and after diagnosis of multiple sclerosis. Mult Scler 2016; 22: 1859–1866. 27650517 10.1177/1352458516667567

[11] Brundin L Kobelt G Berg J, et al; European Multiple Sclerosis Platform. New insights into the burden and costs of multiple sclerosis in Europe: results for Sweden. Mult Scler 2017; 23: 179–191. 28643601 10.1177/1352458517708682

[12] Gyllensten H Wiberg M Alexanderson K, et al How does work disability of patients with MS develop before and after diagnosis? A nationwide cohort study with a reference group. BMJ Open 2016; 6: e012731. 10.1136/bmjopen-2016-012731 PMC5128990 27856477

[13] Hillert J Stawiarz L. The Swedish MS registry – clinical support tool and scientific resource. Acta Neurol Scand 2015; 132: 11–19. 26046553 10.1111/ane.12425 PMC4657484

[14] Svenska neuroregister n.d. The Swedish Neuroregistry, http://www.neuroreg.se/

[15] Background Facts, Labour and Education Statistics 2011 4, Integrated database for labour market research, www.scb.se/statistik/_publikationer/AM9901_1990I09_BR_AM76BR1104.pdf (2011, accessed 13 February 2019).

[16] Kavaliunas A Wiberg M Tinghog P, et al Earnings and financial compensation from social security systems correlate strongly with disability for multiple sclerosis patients. PLoS One 2015; 10: e0145435. 26695832 10.1371/journal.pone.0145435 PMC4691204

[17] Wiberg M Friberg E Stenbeck M, et al Sources and level of income among individuals with multiple sclerosis compared to the general population: a nationwide population-based study. Mult Scler 2015; 21: 1730–1741. 25698166 10.1177/1352458515570767

[18] Landfeldt E Castelo-Branco A Svedbom A, et al The long-term impact of early treatment of multiple sclerosis on the risk of disability pension. J Neurol 2018; 265: 701–707. 29392457 10.1007/s00415-018-8764-4 PMC5834562

[19] Jennum P Wanscher B Frederiksen J, et al The socioeconomic consequences of multiple sclerosis: a controlled national study. Eur Neuropsychopharmacol 2012; 22: 36–43. 21669514 10.1016/j.euroneuro.2011.05.001

[20] Pearson JF Alla S Clarke G, et al Multiple sclerosis impact on employment and income in New Zealand. Acta Neurol Scand 2017; 136: 223–232. 27891575 10.1111/ane.12714

[21] Pfleger CC Flachs EM Koch-Henriksen N. Social consequences of multiple sclerosis (1): early pension and temporary unemployment – a historical prospective cohort study. Mult Scler 2010; 16: 121–126. 20007430 10.1177/1352458509352196

[22] Kingwell E Marriott JJ Jette N, et al Incidence and prevalence of multiple sclerosis in Europe: a systematic review. BMC Neurol 2013; 13: 128. 24070256 10.1186/1471-2377-13-128 PMC3856596

[23] Dunn SE Gunde E Lee H. Sex-based differences in multiple sclerosis (MS): part II: rising incidence of multiple sclerosis in women and the vulnerability of men to progression of this disease. Curr Top Behav Neurosci 2015; 26: 57–86. 25690592 10.1007/7854_2015_370

[24] Tinghog P Hillert J Kjeldgard L, et al High prevalence of sickness absence and disability pension among multiple sclerosis patients: a nationwide population-based study. Mult Scler 2013; 19: 1923–1930. 23652218 10.1177/1352458513488234

[25] Pfleger CC Flachs EM Koch-Henriksen N. Social consequences of multiple sclerosis: clinical and demographic predictors – a historical prospective cohort study. Eur J Neurol 2010; 17: 1346–1351. 20402759 10.1111/j.1468-1331.2010.03020.x

[26] Bowles S Gintis H Osborne M. The determinants of earnings: skills, preferences, and schooling. Amherst, MA: University of Massachusetts, 2000.

[27] Alping P Piehl F Langer-Gould A, et al; COMBAT-MS Study Group. Validation of the Swedish multiple sclerosis register: further improving a resource for pharmacoepidemiologic evaluations. Epidemiology 2019; 30: 230–233. 30721167 10.1097/EDE.0000000000000948 PMC6369893

[28] Sormani MP Bruzzi P. Can we measure long-term treatment effects in multiple sclerosis? Nat Rev Neurol 2015; 11: 176–182. 25534913 10.1038/nrneurol.2014.237

[29] Kavaliunas A Manouchehrinia A Danylaite Karrenbauer V, et al Income in multiple sclerosis patients with different disease phenotypes. PLoS One 2017; 12: e0169460. 28081163 10.1371/journal.pone.0169460 PMC5231357

[30] Castelo-Branco A Landfeldt E Svedbom A, et al Clinical course of multiple sclerosis and labor-force absenteeism: a longitudinal population-based study. Eur J Neurol 2019; 26: 603–609. 30414299 10.1111/ene.13863

[31] Kavaliunas A Danylaite Karrenbauer V Gyllensten H, et al Cognitive function is a major determinant of income among multiple sclerosis patients in Sweden acting independently from physical disability. Mult Scler 2019; 25: 104. 29143553 10.1177/1352458517740212

