# Supplementary figures and images for: Importance of early treatment decisions on future income of multiple sclerosis patients
Source: Mult Scler J Exp Transl Clin. 2020 Oct 7;6(4):2055217320959116. doi: 10.1177/2055217320959116 (PMC7564625; doi:10.1177/2055217320959116)

Kaplan-Meier survival estimates

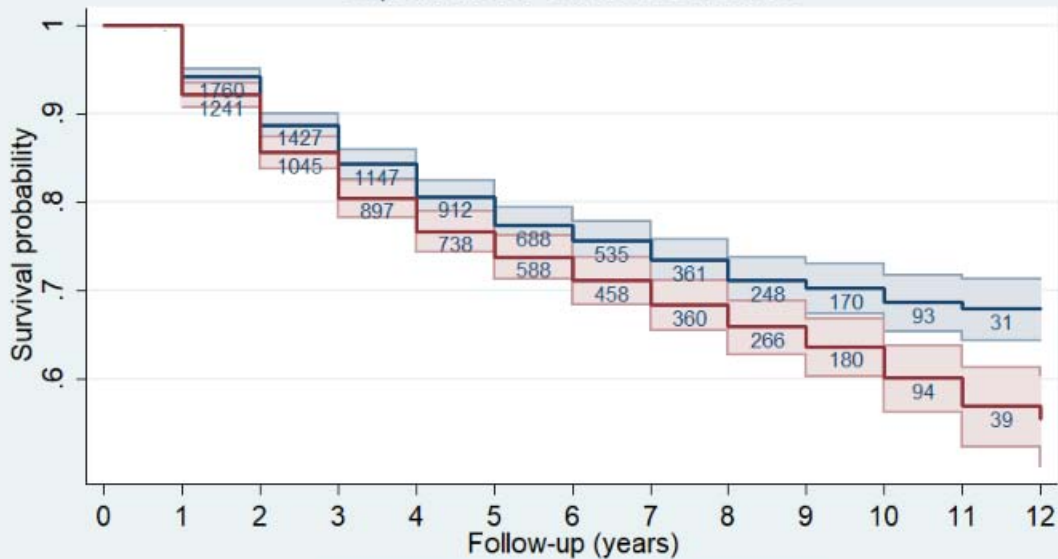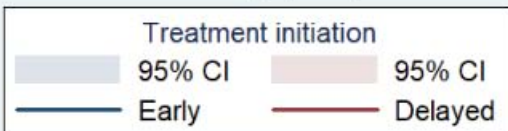

Supplement: sj-pdf-1-mso-10.1177_2055217320959116 - Supplemental material for Importance of early treatment decisions on future income of multiple sclerosis patients [file sj-pdf-1-mso-10.1177_2055217320959116.pdf]
