# Supplementary material for: Importance of early treatment decisions on future income of multiple sclerosis patients
Source: Mult Scler J Exp Transl Clin. 2020 Oct 7;6(4):2055217320959116. doi: 10.1177/2055217320959116 (PMC7564625; doi:10.1177/2055217320959116)
Supplement: sj-pdf-2-mso-10.1177_2055217320959116 - Supplemental material for Importance of early treatment decisions on future income of multiple sclerosis patients [file sj-pdf-2-mso-10.1177_2055217320959116.pdf]

Supplementary Table 1. Crude hazard ratios to lose earnings

Covariate	HR	SE	p	95% CI	
Time to treatment:					
≤2 years	Ref.				
>2 years	1.27	0.09	0.001	1.11	1.45
Sex:					
Males	Ref.				
Females	1.03	0.08	0.68	0.89	1.20
Age at onset:					
<50 years	Ref.				
≥50 years	1.97	0.24	<0.001	1.55	2.51
Education:					
Higher	Ref.				
Secondary	1.93	0.15	<0.001	1.65	2.26
Lower	3.24	0.36	<0.001	2.62	4.02
Family situation:					
Married/cohabitant	Ref.				
Single	1.27	0.09	<0.001	1.11	1.45
Country of birth:					
EU and Norway	Ref.				
Other	1.70	0.22	<0.001	1.32	2.21
Type of living area:					
Larger cities	Ref.				
Medium-sized municipalities	0.94	0.08	0.45	0.80	1.10
Smaller municipalities	1.12	0.10	0.20	0.94	1.32
Baseline EDSS:					
0-1.5	Ref.				
2-4.5	1.76	0.13	<0.001	1.52	2.04
≥5	4.82	0.53	<0.001	3.88	5.98

HR – hazard ratio; SE – standard error; CI – confidence intervals; Ref. – reference; EU – the European Union; EDSS – Expanded Disability Status Scale.

Supplementary Table 2. Clinical and demographic characteristics of the study population in the analysis of benefits

Patients´ characteristics	All patients	Time to treatment ≤2 years	>2 years	p-value
Number of patients	2975 (100%)	1768 (59%)	1207 (41%)	-
Sex:				0.9*
Males	867 (29%)	514 (29%)	353 (29%)	
Females	2108 (71%)	1252 (71%)	854 (71%)	
Age at MS onset (mean (SD))	31.6 (9.7)	32.9 (9.8)	29.7 (9.3)	<0.001**
Age at treatment initiation (mean (SD))	36.3 (10.3)	33.9 (9.8)	40.0 (10.0)	<0.001**
Baseline EDSS (median (IQR))	1.5 (1.5)	1.5 (2)	2 (2)	<0.001***
Education:				0.2*
Higher	1348 (45%)	784 (44%)	564 (47%)	
Secondary	1356 (46%)	830 (47%)	526 (43%)	
Lower	271 (9%)	154 (9%)	117 (10%)	
Family situation:				<0.001*
Married/cohabitant	1536 (52%)	777 (44%)	759 (63%)	
Single	1439 (48%)	991 (56%)	448 (37%)	
Country of birth:				0.9*
EU and Norway	2789 (94%)	1657 (94%)	1132 (94%)	
Other	186 (6%)	111 (6%)	75 (6%)	
Type of living area:				0.5*
Larger cities	1375 (46%)	815 (46%)	560 (46%)	
Medium-sized municipalities	956 (32%)	595 (34%)	361 (30%)	
Smaller municipalities	644 (22%)	358 (20%)	286 (24%)	
Number of patients who reached the outcome	715 (24%)	352 (20%)	363 (30%)	<0.001*

p-value – for comparisons between two time to treatment groups (≤2 years vs. >2 years). SD – standard deviation. IQR – interquartile range. EDSS – Expanded Disability Status Scale. EU – the European Union.
* Chi-square test; ** one-way ANOVA; *** Kruskal-Wallis test.

Supplementary Table 3. Crude hazard ratios to receive benefits

Covariate	HR	SE	p	95% CI	
Time to treatment:					
≤2 years	Ref.				
>2 years	1.42	0.11	<0.001	1.23	1.65
Sex:					
Males	Ref.				
Females	1.16	0.10	0.08	0.98	1.37
Age at onset:					
<50 years	Ref.				
≥50 years	1.26	0.21	0.17	0.91	1.76
Education:					
Higher	Ref.				
Secondary	1.32	0.11	0.001	1.13	1.55
Lower	1.88	0.22	<0.001	1.48	2.37
Family situation:					
Married/cohabitant	Ref.				
Single	0.92	0.07	0.28	0.80	1.07
Country of birth:					
EU and Norway	Ref.				
Other	1.42	0.20	0.009	1.09	1.88
Type of living area:					
Larger cities	Ref.				
Medium-sized municipalities	1.26	0.11	0.007	1.07	1.49
Smaller municipalities	1.31	0.13	0.005	1.08	1.59
Baseline EDSS:					
0-1.5	Ref.				
2-4.5	2.02	0.16	<0.001	1.73	2.37
≥5	5.07	0.67	<0.001	3.92	6.56

HR – hazard ratio; SE – standard error; CI – confidence intervals; Ref. – reference; EU – the European Union; EDSS – Expanded Disability Status Scale.
